# Health Effects of Ionizing Radiation on the Human Body

**DOI:** 10.3390/medicina60040653

**Published:** 2024-04-18

**Authors:** Jasminka Talapko, Domagoj Talapko, Darko Katalinić, Ivan Kotris, Ivan Erić, Dino Belić, Mila Vasilj Mihaljević, Ana Vasilj, Suzana Erić, Josipa Flam, Sanja Bekić, Suzana Matić, Ivana Škrlec

**Affiliations:** 1 Faculty of Dental Medicine and Health, Josip Juraj Strossmayer University of Osijek, 31000 Osijek, Croatia; 2 Faculty of Electrical Engineering, Computer Science and Information Technology Osijek, Josip Juraj Strossmayer University of Osijek, 31000 Osijek, Croatia; 3 Faculty of Medicine, Josip Juraj Strossmayer University of Osijek, 31000 Osijek, Croatiamilavasilj88@gmail.com (M.V.M.); eric.suzana@kbo.hr (S.E.); jflam@mefos.hr (J.F.);; 4 General Hospital Vukovar, Županijska 35, 32000 Vukovar, Croatia; 5 Department of Surgery, Osijek University Hospital Center, 31000 Osijek, Croatia; 6 Department of Radiotherapy and Oncology, University Hospital Center Osijek, 31000 Osijek, Croatia; 7 Health Center Vukovar, 32000 Vukovar, Croatia; 8 Health Center Osijek, 31000 Osijek, Croatia; 9 Family Medicine Practice, 31000 Osijek, Croatia

**Keywords:** ionizing radiation, acute radiation syndrome, central nervous system, gastrointestinal system, hematopoietic system, respiratory system, skin, cancer, apoptosis, cell death

## Abstract

Radioactivity is a process in which the nuclei of unstable atoms spontaneously decay, producing other nuclei and releasing energy in the form of ionizing radiation in the form of alpha (α) and beta (β) particles as well as the emission of gamma (γ) electromagnetic waves. People may be exposed to radiation in various forms, as casualties of nuclear accidents, workers in power plants, or while working and using different radiation sources in medicine and health care. Acute radiation syndrome (ARS) occurs in subjects exposed to a very high dose of radiation in a very short period of time. Each form of radiation has a unique pathophysiological effect. Unfortunately, higher organisms—human beings—in the course of evolution have not acquired receptors for the direct “capture” of radiation energy, which is transferred at the level of DNA, cells, tissues, and organs. Radiation in biological systems depends on the amount of absorbed energy and its spatial distribution, particularly depending on the linear energy transfer (LET). Photon radiation with low LET leads to homogeneous energy deposition in the entire tissue volume. On the other hand, radiation with a high LET produces a fast Bragg peak, which generates a low input dose, whereby the penetration depth into the tissue increases with the radiation energy. The consequences are mutations, apoptosis, the development of cancer, and cell death. The most sensitive cells are those that divide intensively—bone marrow cells, digestive tract cells, reproductive cells, and skin cells. The health care system and the public should raise awareness of the consequences of ionizing radiation. Therefore, our aim is to identify the consequences of ARS taking into account radiation damage to the respiratory system, nervous system, hematopoietic system, gastrointestinal tract, and skin.

## 1. Introduction

Ionizing radiation and radioactivity were discovered at the end of the last century. Radiation as a form of environmental pollution began to worry mankind much more after the use of nuclear bombs at the end of the World War II [1]. An increase in the number of countries possessing nuclear weapons or trying to develop them necessarily increases the number of nuclear tests, resulting in massive releases of radioactivity into our surroundings. Additionally, nuclear incidents like those in Chernobyl in 1986 and Fukushima in 2011 clearly show us that no security measures may provide complete protection and safety [2,3,4,5,6]. The use of nuclear energy in civil or military programs, with the consequent accumulation of nuclear waste, also increases the amount of radioactive materials in our environment [7]. Due to the wide use of radiation and radioactivity in health care and science, certain groups of professionals, as well as ordinary people are exposed to the possible harmful effects of radiation [8,9]. Epidemiological data provide no evidence of adverse health effects at doses below 100 mSv; some studies even suggest beneficial effects. There is also increasing evidence that low doses of radiation, such as those used for X-rays or CT scans, may have some health benefits [10]. However, this has yet to be thoroughly researched, and these findings are still the subject of ongoing research and debate. It is worth noting that while some occupationally exposed groups have lower mortality rates than the general population [10], this does not necessarily indicate a causal relationship between radiation exposure and improved health outcomes. Therefore, the present review aims to educate the public about the various consequences of conditions such as acute radiation syndrome (ARS) and other chronic radiation effects. These conditions may be detrimental to health and cause harm to the human body.

## 2. Consequences of Ionizing Radiation to the Human Body

Radioactivity is a natural phenomenon in which the nuclei of unstable atoms spontaneously decay, releasing energy with the emission of alpha particles (α) and beta particles (β), often accompanied by the emission of gamma electromagnetic waves (γ) [11]. Ionizing radiation refers to nuclear radiation consisting of subatomic particles or electromagnetic waves with sufficient energy to ionize atoms or molecules by stripping them of electrons. This category includes gamma rays, X-rays, and the more energetic ultraviolet part of the electromagnetic spectrum. Typical subatomic particles that cause ionization are alpha particles, beta particles, and neutrons. Secondary cosmic particles such as muons, mesons, and positrons are produced when cosmic rays hit the Earth’s atmosphere. In addition, cosmic rays on Earth can produce radioisotopes such as carbon-14, which emit ionizing radiation when they decay [10]. People can be exposed to radiation in various forms. Natural radiation can vary depending on the area, and it has been found that there are high background radiation areas (HBRA) worldwide [12,13]. Most studies investigated the effects of radon and lung cancer in HBRA, and it has been shown that there is no significant increase in cancer incidence in HBRA [14], i.e., no association between long-term, sustained radiation exposure and the incidence of oncologic diseases [15]. In addition to natural sources of radiation, people are also exposed to α, β, and γ radiation resulting from various accidental situations as well as medical diagnostic and therapeutic procedures such as scintigraphy, radiotherapy, computed tomography, and X-ray imaging [16,17]. Due to the strong interaction with the substance it passes through, a single α-particle can store the energy it possesses in only one cell. For this reason, the biological efficiency and the harmful effect of α-radiation are very pronounced [18]. The β-radiation consists of electrons or positrons. Its ionizing effect is much weaker than α-radiation, but its range is greater [19]. The γ-radiation is characterized by the ability to penetrate deeply into the tissue [20], so it can pass through the entire thickness of the human body without significant problems [21].

The amount of the energy of ionizing radiation that a substance absorbs is indicated in Gray (Gy) [22,23]. Under certain conditions, the absorbed dose does not represent a sufficiently precise amount of the harmful effect on the organism. Therefore, an equivalent radiation dose in sievert (Sv) was introduced [24]. A dose of 50 mSv is the dose of occupational exposure and is considered the smallest proven dose that may cause tumor growth if administered over a year [25]. Doses of 2 to 10 Sv received in a short period cause death in 50% of cases, and a dose of 1 Sv received in a short time leads to radiation sickness characterized by nausea, hair loss, and body erythema [26]. Doses greater than 10 Sv, regardless of the exposure time, cause death in a few weeks [27].

The extent of DNA damage caused by ionizing radiation depends primarily on the ionization density, absorbed dose, dose rate, and linear energy transfer (LET). LET is a measure of the locally absorbed energy (kiloelectronvolt, keV) per unit length (micrometer, µm) [28]. In biological systems, radiation depends on the amount of absorbed energy and its spatial distribution, i.e., the LET per unit length covered by an ionizing particle. We distinguish between low and high LET radiation. Photon radiation with low LET (X-rays, beta, or γ-rays, <10 keV/µm) means a homogeneous energy deposition in the entire tissue volume. In contrast, high LET radiation (protons, alpha particles, and neutrons, >10 keV/µm) decelerates faster than photons, resulting in the formation of a fast Bragg peak, which produces a low entrance dose with a greater depth of tissue penetration that increases with radiation energy [29]. A wide range of DNA lesions can occur during radiation treatment. The most damaging lesions are double breaks, i.e., breaks of both DNA strands and oxidative base lesions, which damage and kill cancer cells, leading to an adequate therapeutic effect of radiotherapy [30]. The main characteristics of ionizing radiation are the direct or indirect creation of DNA breaks and leading the cell into apoptosis and cell death [31]. Radiation has a direct biological effect when it acts on a DNA molecule containing genetic information for a specific biological development or a cellular component necessary for the cell’s survival. Improper DNA repair can lead to mutations or chromosomal abnormalities [32,33]. Indirect DNA breakage occurs when low energy rays enter the cell and electrons hit the water molecule, forming hydroxyl ions—free radicals of water [34]. If they are near the DNA, they lead to a break in one of the helices. Depending on the size of the damage to the helix, the cell can repair the damage or enter programmed death [35]. All cells do not divide at the same rate and do not have the same potential to repair [36]. They are most sensitive in the phase of mitosis or the late G2 phase of the cell cycle [37]. Cells that divide faster are more susceptible to damage and respond better to therapy due to less ability to repair the genetic material [38]. Both low and high LET radiation have a direct or indirect effect on DNA. Direct effects are caused by the direct ionization and excitation of DNA molecules, which disrupt the molecular structure [39]. In contrast, indirect effects are mediated by the radiolysis of water, whereby free radicals that act as mediators and cause DNA damage are formed. Approximately two-thirds of radiation-induced DNA damage is caused by the indirect effects of reactive oxygen species (ROS) [40]. High-LET radiation is more lethal than comparable doses of low-LET radiation due to a condensed energy deposition pattern and a very dense ionization pattern that causes potentially lethal DNA damage [29].

FLASH radiation is about a thousand times faster than conventional radiation; it can interrupt radiochemical reactions and thus disrupt biological processes. FLASH radiotherapy (FLASH RT) is based on the tissue-sparing effects of ultra-high dose rate (UHDR) radiotherapy. In order to understand the FLASH effect, it is important to know how cells react to DNA damage [41]. FLASH RT is currently being tested as an innovative cancer treatment in clinical research. It delivers an ultra-high radiation dose to the tumor in the shortest possible time. It spares normal tissue from radiation-related side effects such as skin toxicity, gastrointestinal complications, and damage to organs at risk [42].

Radiation biology makes an important contribution to understanding the consequences of ionizing radiation. It explains their effects on targeted and non-targeted cells, tissues, and organs, which can have a late impact on the development of primary and secondary cancers. In non-irradiated tissues, a non-target effect (bystander effect) may occur due to the release of clastogenic factors from irradiated cells. The secretion of clastogenic factors results from activating signaling pathways caused by DNA damage and cell death, i.e., epigenetic dysfunction. Consequently, the resulting oxidative stress affects the secretion of miRNAs and exosomes and stimulates signaling pathways that can lead to epigenetic modulation and radiation-induced carcinogenesis [43]. Radioadaptation is a phenomenon related to the adaptive response of the cell to the effect of the first dose of radiation. It refers to the faster recovery of damaged DNA and the reduction in the frequency of its mutations after applying a low initial dose and before applying a highly damaging dose. Based on the studies, it was found that the strongest radioadaptation and the weakest cellular radiosensitivity correlate with each other [44]. The phenomenon of radiation adaptive response is, therefore, important for the recovery of irradiated cells and should be taken into account in radiobiological studies at low doses [45]. High doses of ionizing radiation are often used as a therapeutic strategy to destroy malignant cells (cancer cells). This therapy has extraordinary clinical significance. An obstacle to effective treatment can be resistance to radiation, known as radioresistance. In order to solve this problem, it is necessary to clarify the pathways and molecular mechanisms of the development of radioresistance [46].

When treating cancer patients, the radiation affects not only the rapidly proliferating tumor cells but also normal tissue and other nearby organs that are at risk because they are in the planned radiation field [47]. The extent of damage to healthy tissue by ionizing radiation depends on the radiosensitivity of individual cells, the radiation dose, the size of the fraction, and the volume treated [48]. One of the most important late effects of RT, which affects the patient’s quality of life and causes considerable morbidity, is radiation fibrosis syndrome (RFS), which can occur in the gastrointestinal and urogenital tracts, skin, subcutaneous tissue, and respiratory system, as well as in any other organ within the radiation field [49,50]. Radiation damage increases inflammation and stimulates the production of myofibroblasts from differentiated fibroblasts, leading to the production of excess collagen and various components of the extracellular matrix. This process is supported by a reduced secretion of remodeling enzymes [51]. The subsequent fibrosis reduces tissue compliance and causes cosmetic and functional impairments that significantly affect the quality of life of most cancer patients, especially those with head and neck cancer [52].

### 2.1. Effects of Radiation on the Respiratory System

Exposure to radioactive radiation may have severe consequences on the respiratory system [53]. The impact varies based on factors such as the amount of radiation received, the length of exposure, and the specific type of radiation encountered [54]. Some potential consequences are ARS, radiation pneumonitis, increased risk of lung cancer, respiratory infections, and long-term respiratory effects [55]. 

ARS, alternatively referred to as radiation toxicity or radiation sickness, is an acute illness resulting from the exposure of the entire body (or a significant portion of it) to a high dose of penetrating radiation within a brief timeframe (typically measured in minutes) [56]. The essential requirements for ARS include a substantial radiation dose exceeding 0.7 Gy. The dose is usually externally received, meaning the radiation source originates outside the individual’s body. Furthermore, the radiation, such as high-energy X-rays, γ-rays, and neutrons, must be penetrating. It necessitates the involvement of the entire body, and the dose must have been administered over a short duration. The four stages of ARS are as follows (Table 1): (1). Prodromal stage—individuals experience classic symptoms such as vomiting, nausea, anorexia, and possibly diarrhea (depending on the dose). The respiratory manifestation in this stage is respiratory distress [57]. These symptoms commonly manifest within a timeframe ranging from minutes to days after exposure and can intermittently persist for minutes to several days [58]. (2). Latent stage—characterized by a period of apparent well-being. During this stage, the individual may outwardly appear and feel relatively healthy despite being internally affected by radiation. This phase can have a duration of a few hours or extend to several weeks [59]. Respiratory manifestations are coughing (radiation exposure can irritate the respiratory system, leading to persistent coughing; the cough may be dry or productive), shortness of breath (radiation-induced damage to the lungs can cause difficulty in breathing, resulting in shortness of breath or breathlessness), chest pain (in some cases, radiation exposure may cause chest discomfort or pain, which can result from inflammation or damage to the lungs or surrounding tissues), wheezing (radiation-induced inflammation and narrowing of the airways) characterized by a whistling or rattling sound during breathing), and pulmonary edema (severe radiation exposure can cause fluid accumulation in the lungs, leading to pulmonary edema). Symptoms may include severe shortness of breath, coughing up frothy sputum, and a sense of drowning. (3). Manifest illness stage—the symptoms experienced by the patient depend on the specific syndrome (symptoms can persist for varying durations, ranging from a few hours to several months). (4). Recovery or death—the prognosis is grim for those who do not recover. Most patients who do not survive will succumb to the effects of ARS within several months of exposure. The process of recovery, for those who can recover, can span from several weeks to years. 

Radiation pneumonitis is acute lung tissue inflammation due to exposure to radiation (also common among patients who have received radiation therapy) [60]. Common clinical symptoms observed during this stage usually include a cough, difficulty breathing/shortness of breath (dyspnea), and low-grade fever. The severity of lung injury can significantly vary among individuals exposed to radiation. Radiation pneumonitis severity is classified according to clinical manifestations. The grading scale is as follows: Grade 1 (characterized by mild symptoms such as a dry cough that occurs during exertion), Grade 2 (involves persistent coughing necessitating the use of narcotic anti-tussive agents and/or experiencing difficulty breathing/shortness of breath with minimal exertion, but not at rest), Grade 3 (presents with severe coughing, unresponsive to narcotic agents, and/or dyspnea at rest), Grade 4 (indicates a critical condition of respiratory insufficiency that demands oxygen therapy or assisted ventilation) and Grade 5 (denotes the most severe outcome, resulting in death) [61]. Late radiation-induced lung injury typically presents as pulmonary fibrosis/radiation fibrosis. Radiographic evidence of the findings related to radiation-induced pulmonary fibrosis normally becomes visible around six months after exposure. Nearly all patients who develop this condition show evidence of fibrosis within 24 months following radiation exposure. It is worth noting that individuals who experience radiation pneumonitis are more prone to developing radiation-associated fibrosis. 

Exposure to ionizing radiation, such as certain types of radioactive particles, may increase the risk of developing lung cancer [62]. Lung cancer caused by radiation exposure may develop years or even decades after the initial exposure [63]. Some mathematical models of radionuclide depositions show that when radioactive elements are inhaled into the human respiratory system, they can hit the epithelial walls of large airways and cause the induction of squamous cell carcinoma and small-cell carcinoma. Particles that reach the bronchiolar epithelium and alveolar-interstitial tissues (small airways) have the potential to initiate the development of certain types of lung cancer, such as large-cell carcinoma and adenocarcinoma [64]. The digression of the topic is radiation-induced lung cancer as a potential long-term complication of chest radiotherapy [65]. Aside from lung cancer, several other malignancies may potentially be induced in chest radiotherapy patients [66]. These include sarcomas, osteosarcomas (the most common type from irradiated bones), and malignant fibrous histiocytomas that typically arise from soft tissues [67]. Breast cancer, pleural mesothelioma, and esophageal cancer are also among the potential malignancies that can be induced due to chest radiotherapy [67].

Radiation exposure may weaken the immune system and lead to damage to the normal function of alveolar epithelium [68], making individuals more susceptible to respiratory infections such as pneumonia. These infections can further compromise the respiratory system and lead to additional complications. In studies that analyzed the airway microbiome analysis in people receiving radiotherapy, the conclusion was that *Escherichia*, *Lactobacillus*, *Parabacteroides*, *Shigella*, and *Bifidobacterium* were found to be more abundant (these observations have the potential to serve as novel bacterial biomarkers) [69]. However, low radiation exposure levels may sometimes strengthen the immune system due to radioresistance or adaptive response. However, this effect is not universal and depends on various factors [70,71,72,73]. 

Chronic radiation exposure may have long-term effects on the respiratory system and bronchopulmonary diseases, especially chronic obstructive pulmonary disease (COPD) [74]. Symptoms include chronic cough, shortness of breath, and reduced lung capacity [62]. These effects may persist for years after exposure and may significantly impact the quality of life [75]. 

### 2.2. The Effect of Radiation on the Nervous System

Until recently, it seemed that nervous system tissue was radiation-immune. However, in recent years, there has been growing evidence that the nervous system responds to even low radiation doses and that some damage frequently accompanies this reaction. Ionizing radiation’s harmful effects on the central nervous system (CNS) are a severe outcome of the fields of cancer radiotherapy and space exploration [20,76]. Direct proof of radiation’s harmful impacts on the CNS can be found in atomic bomb survivors and Chernobyl accident victims. Survivors experience mental health problems, memory impairments, and cognitive impairments; a few have abnormalities in electroencephalographic patterns [20]. Ionizing radiation (IR) exposure is every day and can be artificial or natural. On average, there is 3.0 mSv of exposure per person per year worldwide, of which 2.4 mSv come from natural sources and 0.6 mSv come from artificial sources [77,78].

While most of the population is exposed to relatively low levels, some people may be exposed to higher levels of IR due to their environment, occupation, lifestyle, and needs [79]. The CNS is more vulnerable to metabolic stress than other tissue types, and ionizing radiation particles have the physical capacity to produce free radicals that may result in direct or indirect DNA damage [80]. Genomic stability is maintained with the help of DNA damage repair mechanisms. However, some lesions never recover, and the buildup of these lesions may ultimately cause cell death by apoptosis or autophagy. Radiation diseases such as brain tumors may eventually appear if the damage does not result in cell death [81]. Individual factors like age, sex, other medical diagnoses, psychological factors, and genetics can also impact a patient’s CNS dysfunction after irradiation [82]. The daily functioning and quality of life can be significantly affected by radiation injury of the CNS. While brain tissue can tolerate high doses when given in small volumes, giving low doses in large volumes results in late effects [83]. The brain and spinal cord are late-responding tissues [84]. A single total body exposure to a high dose of ionizing radiation, 10 Gy or more, is acutely lethal, as demonstrated by the nuclear disasters of Hiroshima and Nagasaki. After such exposure, cerebral vascular syndrome can develop hours later. Extreme cerebral edema caused by vascular injury results in headache, nausea, seizures, herniation, and eventual death. In contrast, fractionated whole-brain radiation therapy for brain metastases at doses of 30 Gy is well tolerated in the short-term but causes fatigue and alopecia [84]. The determination of cell fate and the decline in neurogenesis are influenced by cellular microenvironment changes [85]. Oxidative stress from radiation activates proinflammatory pathways and increases the quantity of activated microglia, which are potent neurogenesis inhibitors [86,87]. The death of endothelial cells that results in thrombus formation on the exposed matrix and small vessel occlusion can also be brought on by radiation. After radiation, atherosclerosis and microangiopathy can cause vascular insufficiency and infarction [88]. 

Symptoms can be temporary or permanent, and radiation injury may be acute or delayed. Demyelination and edema are the leading causes of acute radiation injury. White matter changes and neuronal loss are late effects [84]. Within two weeks of radiation, acute encephalopathy develops due to a temporary fluid buildup within the brain’s cells, resulting in cerebral edema [89,90]. Some symptoms include confusion, drowsiness, nausea, vomiting, and headaches. Dose per fraction, which is typically >3 Gy/fraction, is the leading risk factor [89,91]. Early-delayed radiation encephalopathy could start two weeks to 6 months after the end of radiation therapy. The pathophysiology is most likely temporary demyelination brought on by oligodendroglial injury and blood-brain barrier disruption. The signs and symptoms resemble those of acute encephalopathy. The diagnosis is clinical since neither the imaging nor the EEG reveals distinct changes. Within a few weeks, patients get better [89,91]. Usually, in patients with glioblastoma, six weeks after radiotherapy, pseudoprogression could occur. Up to 6 months after treatment, some patients may experience a worsening of their pre-existing neurological deficits [92,93]. At the same time, brainstem syndrome occurs between 1 and 3 months after radiation therapy with symptoms of ataxia, dysarthria, and nystagmus with hearing loss [89]. Usually, 6 to 12 months after radiation, late complications appear. Radionecrosis (RN) and late-delayed radiation encephalopathy are the two main complications. The most severe side effects of radiotherapy seem to be RN. From a pathophysiology perspective, tissue death is caused by a combination of direct glial injury, endothelial cell damage, and blood-brain barrier (BBB) damage. Usually, it happens years after radiation. The total radiation dose and fraction size are the most important risk factors [89,94]. Delayed cognitive impairment brought on by late-delayed radiation encephalopathy can range from mild memory loss to severe dementia. Sometimes using anticholinesterases for symptoms could help [89]. 

Because irradiated patients are more likely than the general population to develop secondary brain and spine tumors, radiation may cause radiation-induced brain tumors. The most frequent tumors are meningiomas, which occur 70% of the time. Gliomas occur 20% of the time, and sarcomas occur 10% of the time [89]. Childhood IR exposure of moderate to high doses > 0.5 Gy is a known risk factor for CNS cancers. Some studies suggested a dose-response relationship between exposure to ionizing radiation and the risk of CNS tumors, whereas other studies found no evidence for such a relationship [79]. Four risks that NASA has highlighted may indicate significant health issues for astronauts, including carcinogenesis, degenerative tissue changes, CNS performance decline, and acute radiation syndrome [20]. Acute and chronic CNS radiation cause a complex network of molecular and cellular changes, such as DNA damage, oxidative stress, cell death, and systemic inflammation. These changes affect synaptic plasticity and neuronal structure, impacting behavior and cognition [76].

### 2.3. Hematopoietic Syndrome

With 1–8 Gy irradiation, the clinical form of ARS occurs already in the latent phase. It turns into a hematopoietic syndrome resulting from stem cell damage in the bone marrow and lymphatic tissue [95]. A slight drop in the number of blood cells also occurs with lower doses of radiation [96]. There are fundamental changes in the blood count with marked granulocytopenia, thrombocytopenia, and immune system weakening [26]. A total decrease in hematopoietic cells occurs, especially in the bone marrow [97]. Namely, an increase in the number of adipose cells (mesenchymal material cells) in the cavities of the bone marrow occurs because adipose cells are less sensitive than hematopoietic stem cells. In this way, the structure and quality of the bone are significantly impaired [98]. Bone marrow contains two lines of multipurpose stem cells: hematopoietic (HSC) and mesenchymal stem cells (MSC) [99]. In principle, HSCs are controlled by immune cell mechanisms [100], and MSCs produce cartilage, bones, muscle tendons, ligaments, and adipose cells [101]. HSCs give rise to the myeloid and lymphoid lineages; myeloid cells give rise to neutrophils, eosinophils, basophils, monocytes, macrophages, platelets, and erythrocytes, and lymphoid cells give rise to T-cells, B-cells, and natural killer (NK) cells [102]. The next stage of bone marrow failure is the inability to produce blood cells (especially those needed to control the immune status) [103]. Thus, the acquired disorder caused by ionized cells consequently includes aplastic anemia, hypoplastic myelodysplastic syndrome, developed aplasia of red blood cells, megakaryocytic thrombocytopenia, and chronic neutropenia [104]. 

Therefore, the influence of ionizing radiation has a threatening effect on the immune health of irradiated persons (bone marrow, thymus, and spleen), so from experience so far, it is considered that the said influences are crucial in the clinical sense because they consequently lead to a sudden decline in vital functions (anemia, leukemia, secondary inflammation, immune insufficiency, loss of bone mass, etc.) [105]. Since the effects of the hematopoietic syndrome in patients with ARS are cumulative with other syndromes (gastrointestinal, neurovascular, and dermatological), we consider them the leading cause of the challenging recovery of irradiated patients and even death [58]. 

### 2.4. The Effect of Radiation on the Gastrointestinal Tract

Gastrointestinal acute radiation syndrome is characterized by significant intestinal dysfunction brought on by large doses of radiation (6–15 Gy) [106]. Radiation impacts the cells lining the digestive tract, resulting in gastrointestinal syndrome. After exposure to radiation of at least 6 Gy, severe nausea, vomiting, and diarrhea may occur in less than an hour, and the symptoms may cause severe dehydration. However, they usually go away after two days. People feel well over the next 4 to 5 days (latent phase), but the digestive tract’s lining cells, which typically serve as a protective barrier, begin to degrade and die. After that, severe diarrhea, often bloody, returns, which causes dehydration once more. The body can become infected by bacteria from the digestive tract, leading to sepsis. People who have received so much radiation also develop hematopoietic syndrome, which leads to bleeding and infections and increases the risk of death. Death is frequently the result when exposed to radiation of 6 Gy or more. However, roughly 50% of people can live with modern medical assistance. The Radiation Therapy Oncology Group (RTOG) and the European Organization for Research and Treatment of Cancer (EORTC) scale proposed a gastrointestinal severity scoring system to assess radiation-induced gastrointestinal injury, to save individuals from potential gastrointestinal acute radiation syndrome injury [107].

Along with chemotherapy and surgery, radiotherapy is one of the main therapeutic methods of cancer treatment. Although radiotherapy affects cancer cells, it inevitably damages surrounding cells and tissues. This especially happens in thoracic and abdominal radiotherapy [108]. Radiation can more easily harm body organs with rapid cell division, such as the intestines and bone marrow, than organs with slower cell division, like the muscles and tendons. One of the most sensitive organs in the body is the digestive system, which comprises cells that grow and differentiate quickly. High doses of ionizing radiation can produce a variety of reactive oxygen species (ROS) and reactive nitrogen species (RNS), including radicals, which can have adverse effects such as ulceration, discomfort, nausea, vomiting, diarrhea, and malnutrition [109]. The digestive system is, therefore, one of the most susceptible physiological systems to radiation therapy and typically experiences the most severe side effects of radiotherapy. The digestive system comprises the digestive tract and auxiliary digestive organs (mouth, salivary glands, pancreas, liver, and gall bladder) [110]. 

Two types of digestive radiation injuries can occur during radiotherapy: digestive tract injuries and salivary gland injuries [108]. Hyposalivation is a direct result of radiation-induced salivary gland damage. Therefore, a lack of saliva results in xerostomia, mucositis, nutrient deficits, oral infections, and functional abnormalities (such as difficulty chewing, dysphagia, and taste loss) [111]. Radiation harm in other parts of the gastrointestinal tract, including the esophagus, stomach, intestines, and anus, starts with mucosal inflammation and progresses to diarrhea, constipation, and bleeding [110]. In addition, radiation causes hereditary genotoxicity and degrades genetic material. Therefore, creating strategies to counter radiation harm is believed to be crucial [112]. A conventional method of preventing radiation-related damage is to protect the target area’s sensitive areas. Visceral organ defense, however, is challenging [113,114]. Hydrogels are the solution to this issue. Hydrogels are non-toxic three-dimensional cross-linked polymer networks with high water absorption and retention capacity. Hydrogels, like healthy tissue, can readily absorb radiation thanks to this property [108].

Traditional radiation is dose-limited to prevent radiotoxicity to healthy tissues [115]. Fractionated radiation decreases the number of visits and overall cost of treatment without increasing radiotoxicity while increasing the total dose tolerance [116]. Recent research has been done to support the radioprotective properties of the Indian medicinal herb *Podophyllum hexandrum*. These results demonstrate that *Podophyllum* and its constituents/natural chemicals protect the lungs, gastrointestinal tissues, hemopoietic system, and testes by activating DNA repair pathways, inhibiting apoptosis, scavenging free radicals, chelating metals, and activating antioxidant and anti-inflammatory processes [112]. 

Numerous disorders now focus on gut microbiota; studies have also linked it to radiation sensitivity [117]. Since gut microbiota can predict radiation harm, it is conceivable that regulating the gut microbiota could reduce radiation damage. By altering the function of the intestinal barrier, innate immunity, and intestinal repair mechanisms, the microbiota in the intestine significantly contributes to the pathophysiology of radiological injuries [118]. Measures that regulate the gut microbiota include probiotics [119], methionine diet [120], oral gavage with hydrogen water [121], and omega-3 polyunsaturated fatty acids (ω–3 PUFA) [122]. Recently, Guo et al., used fecal grafting. They shared unclean cages to transfer the gut microbiota of human and mouse radiation survivors, and they discovered that *Lachnospiraceae* and *Enterococcaceae* were related to decreased radiation-induced damage. Two metabolites of these two bacteria’s tryptophan pathway provided long-term radioprotection, specifically 1H-indole-3-carboxaldehyde and kynurenic acid. The foundation for the clinical intervention of the human gut microbiota against radiation damage has been laid by this study, which is the first to show the effectiveness of gut microbiota modification in people. These instances demonstrate that the intestinal microbiota offers the potential for diagnosing, establishing prognosis, and managing radiation lesions [123]. A new era of radioprotection might be ushered in by future efforts to restore the ideal microbial composition specific to the patient [124].

Radiation has been shown to cause cell autophagy, cell cycle arrest, and even cell death. Apoptosis and ferroptosis, in particular, should be addressed in radiation-induced digestive injury. Inhibitors of cell death and inflammation may help reduce radiation-induced digestive injury [108,125]. Tetrahydrobiopterin (BH 4), which is created by GTP-cyclohydrolase 1 (Gch1), can be supplemented to treat ionizing radiation-induced vascular endothelial dysfunction and avoid intestinal ischemia. These findings impact the prevention and management of radiation enteritis [126]. The effects of radiation also depend on how much the body is exposed to it. The radiation-induced gastrointestinal injury appears to be a dose-volume effect, meaning that the extent of the lesion is highly dependent on radiation dose and radiation volume [127]. When radiation is beamed to a small region, as in radiation therapy for cancer, it can be administered up to three or four times without seriously harming the body [128]. However, radiation beamed over the whole body surface typically results in death with doses greater than six heats. Another crucial factor is how radiation is distributed throughout the body [129]. High doses of radiation can be employed during radiation therapy for cancer since everything appears to shield the body areas that are more susceptible to the radiation [130]. 

### 2.5. Cutaneous Radiation Injury (CRI)

Cutaneous radiation injury (CRI) involves skin and subcutaneous tissue lesions after radiation exposure [131]. CRI is a type of deterministic effect dependent on dose, type of radiation, irradiated volume, and exposed individuals’ comorbidities [132,133]. These deterministic effects have a threshold of 2 Gy, which means that a certain dose is necessary to produce the effect, after which the effect increases with dose. After radioactive fallout, a primary cause of CRI could be beta particle and gamma ray contact with unprotected skin. Gamma rays with similar energy to the beta particles of 0.4 to 0.6 MeV penetrate 30 cm in soft tissue, whereas β particles would penetrate only 0.1 to 0.2 cm of skin [134]. This is because particles with higher energy require more time to recover their initial energy than particles with lower energy [135].

The first described CRI resulted from WW2 atomic bombs on Hiroshima and Nagasaki in August 1945 [1]. Then, in 1954, in the Marshall Islands in the Pacific, the crew members of a Japanese fishing boat and local inhabitants were exposed to radioactive fallout and sustained severe skin burns. The nuclear plant station incident in Chernobyl 1986 is the most known in history [136]. In everyday medical practice, CRI can result from direct diagnostic radiography and cancer radiotherapy [24]. Radiation skin damage from radiation therapy is often located on the neck, face, and upper body. Approximately 20% to 25% of all patients have more severe symptoms like telangiectasis, skin atrophy, and ulceration [137,138]. CRI does not usually kill a patient unless it interacts with other total-body irradiation (TBI) effects. Still, it can lead to skin necrosis, scarring, severe pain, and skin cancer years after initial exposure [139]. Properly assessing the extent and severity of CRI is often difficult because symptoms can develop over days and weeks after radiation exposure [131]. Therefore, comorbidities, radiation dose, dose rates, evaluation of the surface area in addition to the depth of injury, and TBI must be included in medical, clinical assessment, and surgical decision-making [140,141].

Clinical signs of CRI can vary based on the dose and duration of the exposure [132,138]. Skin is the most affected organ, but if the radiation dose is strong/or the duration is long enough, then even subcutaneous fat tissue or muscle can also be damaged [142]. Clinical presentation can vary from skin erythema (redness), edema (swelling), epilation (hair loss), hyperpigmentation (skin darkening), atrophy (thinning of the skin structure), ulceration (presenting as an open sore), telangiectasia (dilation of small skin vessels), and fibrosis (connective tissue over-production), to necrosis (tissue death due to ischemia) [143]. Radiation dose is the most important factor in CRI progression, which develops over time in four stages (Table 1). The first stage is the prodromal stage, which occurs several hours after exposure and is characterized by early erythema and a sensation of warmth, with an average duration of 1 to 2 days. The second stage is the latent stage, which occurs 1 to 2 days after exposure and has no obvious symptoms. The higher the latent dose, the shorter the latency period; the skin on the face and neck also has a shorter latency period. The third stage is the manifest stage, which occurs days to weeks after exposure and in which redness, edema, and hyperpigmentation are the main symptoms. Skin ulcers and necrosis may also occur depending on the radiation dose. The fourth stage is late effects, which occur weeks and years after exposure if the radiation dose is higher than 10 Gy. Dermal atrophy with a recurrence of skin ulcers is the main symptom.

Pain from radiation to the skin is often resistant to opiates, which can lead to psychological crises for the patients. Therefore, a thorough history and physical examination are essential for diagnosis. Also, a blood count should be performed for the early diagnosis of the hematopoietic syndrome of acute radiation sickness. In addition, ultrasound and thermography are useful for CRI diagnosis [144].

There are currently no drugs specifically cleared to treat CRI, and the treatment should include a variety of medical experts—a multidisciplinary approach. The initial treatment should be carried out in a clean environment, with pain management, infection prevention, anticoagulant prevention, and psychological support. The use of topical corticosteroids, pentoxifylline, in combination with α-tocopherol, provides a synergistic effect [145].

Surgery may be combined with all of the treatments mentioned above. Surgical techniques include wide surgical excision with secondary wound healing, vacuum-assisted wound closure, free flap closure, and local flaps, such as rotation flap, transposition flap, and interpolation flap. On occasion, even amputation is required. In addition, hyperbaric chamber treatments have shown promising results in managing CRI [146].

A new treatment approach was successfully applied in France in 2005 on a construction worker who found a sealed radioactive source in Chile. This treatment was based on dosimetry-guided surgery and the local administration of mesenchymal stem cells derived from bone marrow, providing excellent outcomes [147,148]. Dosimetry-guided surgery can be applied as an early procedure when deep ulceration and necrosis can be expected (tissue radiation dose >25 Gy) [148]. MSCs are injected during surgery and in several sessions following surgery to deliver paracrine factors like anti-inflammatory cytokines, growth factors, and microvesicles that contribute to the healing [132]. Unfortunately, medical treatments and follow-ups sometimes take decades because of the nature of cutaneous radiation injury [131,132].

## 3. Conclusions

Ionizing radiation is an important scientific discovery of the last two centuries and is indispensable for modern life. It is also of crucial importance in the treatment of cancer patients, where protective measures against the undesirable effects of ionizing radiation are essential. The interaction between radiation and genes must be further researched to better understand the mechanisms of radiation and develop better ways of protecting against it. In order to deal efficiently with acute radiation syndrome, it is necessary to comply with legal regulations, to carry out constant professional monitoring and control of radiation sources, and to constantly train the medical staff who handle them and to take appropriate protective measures.

## Figures and Tables

**Table 1:** Stages of acute radiation syndrome in different organ systems.

	Prodromal Stage (Hours after Exposure)	Latent Stage (1–2 Days after Exposure)	Manifest Stage (Days to Weeks after Exposure)	Late Effects (Weeks and Years after Exposure)
Respiratory manifestation	Respiratory distress	Cough Shortness of breath Chest pain Wheezing Pulmonary edema	Radiation pneumonitis Respiratory infections	Pulmonary fibrosis COPD Lung cancer
Nervous system	Acute encephalopathy (headaches, nausea, vomiting, drowsiness, confusion, ataxia)	Encephalopathy Radiation myelopathy Damaged blood-brain barrier Interstitial edema Acute inflammation Petechial hemorrhages Meningitis Hypertrophy of perivascular astrocytes	Encephalopathy (headaches, nausea, vomiting, drowsiness, confusion, ataxia)	Ataxia Dysarthria Nystagmus with hearing loss Radionecrosis Radiation-induced brain tumors Coma
Hematopoietic system		Lymphopenia	Neutropenia Thrombocytopenia	Anemia Pancytopenia Leukemia Secondary inflammation Immune insufficiency Loss of bone mass Hypoplasia or aplasia of bone marrow
Gastrointestinal tract	Vomiting Nausea Anorexia Diarrhea Cramps	Dehydration Anorexia	Malaise Anorexia Severe diarrhea Fever Dehydration Electrolyte imbalance Malnutrition Malabsorption	Infection Sepsis Necrosis of the bowel wall Stenosis Ileus Perforation
Cutaneous radiation injury	Erythema Heat sensation	Redness Blisters Ulceration	Erythema Edema Hyperpigmentation Ulceration Necrosis	Dermal atrophy with ulcers Telangiectasia Local edema Connective tissue fibrosis Skin cancer

## Data Availability

Not applicable.
